# Managing patients taking edoxaban in dentistry

**DOI:** 10.4317/jced.53431

**Published:** 2017-02-01

**Authors:** Adrian Curto, Daniel Curto, Jorge Sanchez

**Affiliations:** 1 Proffesor in Pediatric Dentistry. Faculty of Medicine, University of Salamanca, Salamanca, Spain; 2 Student in Medicine. University of Salamanca, Salamanca, Spain; 3 Master in Oral Surgery. Alfonso X El Sabio University, Madrid, Spain

## Abstract

**Background:**

Anticoagulation therapy is used in several conditions to prevent or treat thromboembolism. A new group of oral anticoagulants with clear advantages over classic dicoumarin oral anticoagulants (warfarin and acenocoumarol) has been developed in recent years. The Food and Drug Administration has approved edoxaban, dabigatran, rivaroxaban and apixaban. Their advantages include: predictable pharmacokinetics, drug interactions and limited food, rapid onset of action and short half-life. However, they lack a specific reversal agent.

**Material and Methods:**

This paper examines the available evidence regarding rivaroxaban and sets out proposals for clinical guidance of dental practitioners treating these patients in primary dental care. A literature search was conducted through July 2016 for publications in PubMed and Cochrane Library using the keywords “edoxaban”, “dabigatran”, “rivaroxaban”, “apixaban”, “new oral anticoagulants”, “novel oral anticoagulants”, “bleeding” and “dental treatment” with the “and” boolean operator in the last 10 years.

**Results:**

The number of patients taking edoxaban is increasing. There is no need for regular coagulation monitoring of patients on edoxaban therapy. For patients requiring minor oral surgery procedures, interruption of edoxaban is not generally necessary. Management of patients on anticoagulation therapy requires that dentists can accurately assess the patient prior to dental treatments.

**Conclusions:**

Their increased use means that oral care clinicians should have a sound understanding of the mechanism of action, pharmacology, reversal strategies and management of bleeding in patients taking edoxaban. There is a need for further clinical studies in order to establish more evidence-based guidelines for dental patients requiring edoxaban.

** Key words:**Edoxaban, dabigatran, rivaroxaban, apixaban, novel oral anticoagulants, bleeding.

## Introduction

There are a large number of patients being treated with oral anticoagulants and antiplatelet drugs. Oral anticoagulation and prevention of venous thromboembolism has been possible for decades thanks to the use of vitamin K antagonists, mainly warfarin and acenocoumarol (1).

Vitamin K antagonist oral anticoagulants have a narrow therapeutic window, requiring regular International Normalized Ratio (INR) monitoring, and they also have several drug-drug and drug-food interactions. These disadvantages compromise patients’ quality of life (2).

A great number of medical emergencies as a result of bleeding complications, especially in elderly individuals, are related to the administration of anticoagulant drugs that are vitamin K inhibitors. In the USA, these drugs are the most frequently involved in such emergency hospitalizations (3).

Research to find an alternative to classic oral anticoagulants has been focused on the development of an oral anticoagulant with a wide therapeutic window and little inter- and intra-individual variability that may be administered as a fixed-dose with no need for regular coagulation monitoring and few drug-drug and drug-food interactions. There are currently three direct factor Xa inhibitors (rivaroxaban, apixaban and edoxaban) and one direct thrombin inhibitor (dabigatran) with approved indications for antithrombotic prophylaxis and therapy in different conditions. Among these, the most recently developed and marketed oral anticoagulant is edoxaban.

The ENGAGE AF-TIMI 48 (effective anticoagulation with factor Xa next generation in atrial fibrillation) study is a randomized double-blind clinical trial that assessed the administration of edoxaban vs warfarin on a daily basis in a total of 21,105 patients with nonvalvular atrial fibrillation. The results proved that the efficacy of warfarin for the prevention of stroke and systemic embolism was noninferior to warfarin, and that the administration of edoxaban is associated with less morbidity (lower incidence of bleeding) (4). With regard to the ages of the patients taking part in the study, both for the risk of stroke or systemic embolism, and for major bleeding, the results for both doses of edoxaban were consistent, regardless of this factor (< 75 vs ≥ 75 years of age). In terms of International Normalized Ratio (INR) control, both for the risk of stroke and systemic embolism, and for that of major bleeding, the results were consistent for both doses of edoxaban, regardless of time in the therapeutic range (> 66.4% o ≤ 66.4%), although there was a trend in favour of the high dose of edoxaban for lower risk of major bleeding in patients with poor INR control (5).

Exdoxaban is a direct factor Xa inhibitor with a molecular weight of 548 Dalton. Its protein binding is approximately 54% and its bioavailability is 50%. Its half-life is around 9-11 hours, and peak plasma levels are reached between the first and second hours after administration. Its excretion is 35% renal and the rest hepatic (6).

The Food and Drug Administration (FDA) approved the use of edoxaban, under the name Savaysa®, in January 2015, and in June 2015, the European Medicines Agency (EMA) authorized its marketing under the name Lixiana®. The usual dose is 60 mg every 24 hours, although in patients with renal impairment or low body weight (under 60 kg) the dose should be halved to 30 mg daily. The different cost-effectiveness studies on new oral anticoagulants (edoxaban, dabigatran, rivaroxaban and apixaban) conducted in several countries are fairly consistent, showing that the administration of these drugs seems the best option for patients at increased risk for thromboembolic or bleeding complications and those with poor anticoagulation control with vitamin K antagonists (7).

## Material and Methods

The aim of this paper is to contribute to the discussion on how to approach patients taking edoxaban, before, during and after dental treatments.

In the present contribution we offer an exhaustive review of the literature found in PubMed and Cochrane Library in July 2016. The words used were “edoxaban”, “dabigatran”, “rivaroxaban”, “apixaban”, “new oral anticoagulants”, “novel oral anticoagu-lants”, “bleeding” and “dental treatment” with the “and” boolean operator.

Metaanalyses, systematic reviews, clinical trials and case-control studies were considered. Specialized textbooks were also consulted.

Because of their relatively recent introduction, specific studies regarding dental treatment of patients taking novel oral anticoagulants are available in literature only from 2012.

-Analytical tests

Unlike classic oral anticoagulants that act as vitamin K inhibitors, such as warfarin and acenocoumarol, edoxaban does not require regular coagulation tests. However, there are certain situations where it may be necessary to assess its plasma concentration, such as overdose, bleeding or emergency surgery. There are no significant changes in the results of routine analytical coagulation tests after the administration of edoxaban. Prothrombin time and activated partial prothrombin time change according to plasma edoxaban concentrations, increasing in a dose-dependent manner (8,9).

The best test to assess edoxaban concentrations is the measurement of anti-factor Xa (10,11).

-Drug-drug interactions

New oral anticoagulants in general, and edoxaban in particular, have few drug-drug interactions. Edoxaban is predominantly absorbed in the upper gastrointestinal tract, so that drugs or conditions that increase gastric emptying and intestinal motility may reduce edoxaban dissolution and absorption. Plasma edoxaban concentrations increase when it is combined with ketoconazole, cyclosporine, dronedarone, erythromycin, quinidine or verapamil, and its co-administration with rifampicin leads to a shortened half-life, with possible decreases in its pharmacodynamic effects (12-14).

-Dental management

The prevalence of bleeding complications during invasive dental procedures is higher in patients taking edoxaban and the rest of new oral anticoagulants (15). There are certain dental treatments that require temporary discontinuation of edoxaban before beginning.

There are currently no published studies or protocols in scientific literature on the dental management of patients taking anticoagulants of the type of edoxaban, so that the risk for bleeding should be individually assessed for each patient according to their cardiac and haemorrhagic condition and the bleeding risk involved in the dental procedure to be carried out. While still scarce, there are, however, clinical guidelines published in recent years that describe the perioperative management of patients takin dabigatran, rivaroxaban and apixaban.

Based on the bleeding risk involved in the different dental treatments, they can be classified into procedures with low bleeding risk and procedures with standard and high bleeding risk.

Procedures with low bleeding risk include oral surgery of up to 45 minutes, simple extractions (less than 3 teeth in the same session) and periodontal surgery with low bleeding risk (15-18). According to the studies published on new oral anticoagulants and their dental management, dental procedures with low bleeding risk do not require discontinuation of edoxaban (16-25).

The rest of dental procedures are associated with standard and high bleeding risk, and such cases do require temporary discontinuation of edoxaban. Since there is no scientific basis for discontinuing edoxaban prior to performing dental treatment, when to stop it depends on the pharmacokinetics and pharmacodynamics of the drug and on the patient’s renal function (impaired renal function involves slower clearance of the oral anticoagulant by renal excretion) (20-35).  Table 1 shows the guidelines for edoxa-ban discontinuation.

**Table 1:**
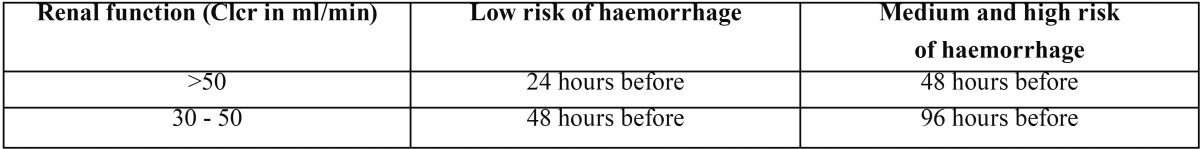
Guide to the suppression of edoxaban.

When to resume edoxaban to re-establish therapeutic anticoagulation depends on the patient’s haemostasis and on the bleeding risk involved in the dental treatment. In cases of oral surgery with standard bleeding risk, edoxaban can be resumed 12-14 hours after the dental treatment (23-37).

The treatment of bleeding after dental procedures should be individualized according to the severity of the bleeding. In cases of mild bleeding, it is enough to use local haemostatic measures such as sutures, gelatine or cellulose sponges and tranexamic acid mouthwashes, which help to reduce the chance of postoperative bleeding (17-40).

## Conclusions

In dentistry and oral surgery, the major concerns in treatment of patients taking oral anticoagulants or antiplatelet drugs is the risk of haemorrhage

There are several novel oral anticoagulant agents (edoxaban, dabigatran, rivaroxaban and apixaban) that are being increasingly used as an alternative to warfarin and acenocoumarol.

For patients requiring simple dental extraction or minor oral surgery procedures, interruption of edoxaban is not generally necessary, while an higher control of bleeding and discontinuation of the drug should be requested before invasive surgical procedures.

The heath professionals have to consider that the number of patients taking edoxaban is increasing. Since available data are not sufficient to establish an evidence-based dental management, the dentist must use caution and attention when treating patients taking edoxaban.
